# The Impact of Ultraviolet Radiation on the Aetiology and Development of Uveal Melanoma

**DOI:** 10.3390/cancers13071700

**Published:** 2021-04-03

**Authors:** Melissa Chalada, Charmaine A. Ramlogan-Steel, Bijay P. Dhungel, Christopher J. Layton, Jason C. Steel

**Affiliations:** 1 School of Health, Medical and Applied Sciences, Central Queensland University, Norman Gardens, QLD 4701, Australia; m.chalada@cqu.edu.au (M.C.); c.ramlogan-steel@cqu.edu.au (C.A.R.-S.); 2 Gene and Stem Cell Therapy Program Centenary Institute, University of Sydney, Camperdown, NSW 2006, Australia; b.dhungel@centenary.org.au; 3 LVF Ophthalmology Research Centre, Translational Research Institute, Woolloongabba, QLD 4102, Australia; 4 Faculty of Medicine, Greenslopes Clinical School, The University of Queensland, Greenslopes, QLD 4120, Australia

**Keywords:** uveal, cutaneous, melanoma, UV, Australia

## Abstract

**Simple Summary:**

Uveal melanoma (UM) is the most common form of eye cancer in adults. It has a poor prognosis and limited treatment options. UM, unlike cutaneous melanoma (CM), historically lacks convincing evidence of causative ultraviolet radiation (UVR) involvement in its pathophysiology and ex vivo experimentation has suggested that UVR cannot even penetrate through the anterior ocular structures to initiate carcinogenic changes. Opposing this idea are the recent publications of UVR damage signatures in UM samples, even in those arising from the posterior uvea. This review provides an up-to-date exploration of endogenous and exogenous factors that can impact ocular susceptibility UVR, whether they form any relationship with UM risk or incidence.

**Abstract:**

Uveal melanoma (UM) is currently classified by the World Health Organisation as a melanoma caused by risk factors other than cumulative solar damage. However, factors relating to ultraviolet radiation (UVR) susceptibility such as light-coloured skin and eyes, propensity to burn, and proximity to the equator, frequently correlate with higher risk of UM. These risk factors echo those of the far more common cutaneous melanoma (CM), which is widely accepted to be caused by excessive UVR exposure, suggesting a role of UVR in the development and progression of a proportion of UM. Indeed, this could mean that countries, such as Australia, with high UVR exposure and the highest incidences of CM would represent a similarly high incidence of UM if UVR exposure is truly involved. Most cases of UM lack the typical genetic mutations that are related to UVR damage, although recent evidence in a small minority of cases has shown otherwise. This review therefore reassesses statistical, environmental, anatomical, and physiological evidence for and against the role of UVR in the aetiology of UM.

## 1. Introduction

The uvea is the vascular layer of the eye and consists of the choroid, ciliary body and iris [1]. The melanocytes in these structures, just like in skin, produce the pigment melanin [2,3]. The quality and quantity of melanin presents as different eye colours and skin colours [4] and provide varying levels of protection from ultraviolet radiation (UVR) [5]. This protection is important because UVR can cause physical or genomic damage to cells [6,7,8]. In their role as light receptors for producing the input for vision, the eyes spend many hours exposed to environmental UVR. Unfortunately, whilst moving the eyes to maximise exposure for visual input, the eye exposes itself to extreme amounts of light radiation and it would be reasonable to suspect that this radiation exposure may put uveal melanocytes at risk of oncogenic transformation into melanoma, especially in individuals genetically predisposed to produce less or lower quality melanin [5]. However, based on traditional evidence and the predominant paradigm, UM is currently classified by the World Health Organisation (WHO) as one of five melanomas triggered by “*risk factors other than cumulative solar damage*” [9,10]. Indeed, it has been demonstrated that wavelengths of the UVR spectrum cannot even penetrate the adult crystalline lens and cornea to reach the posterior eye where the choroid is located [11,12], and yet, the posterior choroid is the most common uveal melanoma origin (85–95%) compared to the iris (3–5% and ciliary body (5–8%) [13]. Despite this, it has recently been demonstrated that a proportion of UMs possess molecular signatures reflective of UVR damage even in those originating from the choroid [6,7,8], suggesting that UVR may, in some cases, be involved in the aetiology of UM.

Australia has often been used as a case study in determining the effects of UVR exposure due to its high Caucasian population, the populations’ enjoyment of the outdoors [14,15] and its proximity to the equator [16,17]. Indeed, UVR exposure has been directly linked to Australia having one of the highest incidences of cutaneous melanoma (CM) in the world [14,15,18], with 60–70% of cutaneous malignant melanomas thought to be caused by solar UVR exposure [19]. Unlike CM, UM is a rare cancer, despite being the most common primary intraocular malignant cancer in adults [13,20]. It has an incidence of only 5 to 10 per million per year in Australia [21] compared to nearly 500 cases of CM per million per year [15]. Regardless, the incidence of UM in Australia is still high compared to Canada (3.75 per million) [22] and the United States (5 per million) [23,24], and is matched only by countries with very high proportions of UVR-susceptible ethnicities (but not necessarily high UVR exposure) such as Denmark (8.6 per million) [25] and Ireland (9.5 per million) [26].

UM resists most current treatments [27,28] with half of the cases [29] eventually resulting in liver metastasis and poor prognosis. It would therefore be ideal if preventative measures can be identified to reduce the risk of developing UM altogether, just as sun-avoidance initiatives have improved the burden of CM in the younger generations [15]. However, despite sharing a common melanocyte origin, the role of UVR in CM is accepted whereas its role in UM remains controversial, even though increased UVR susceptibility is frequently a risk factor for UM (see Table 1). This review seeks to assess the anatomical, physiological, environmental, and statistical evidence for and against a role of UVR in UM induction.

## 2. Interactions between UVR and the Eye

### 2.1. Overview of Radiation Spectrums

The Sun emits a wide range of the electromagnetic spectrum but only a relatively small amount reaches the Earth’s surface, and even less can penetrate the human eye. Wavelengths 400–780 nm, which represent all the colours of the rainbow, are visible to humans [40] while UVR, invisible to humans, consists of UV-C (wavelength 100–280 nm), UV-B (wavelength 280–315 nm), and UV-A (wavelength 315–400 nm) [40]. Due to the presence of the ozone, which absorbs all radiation shorter than 290 nm, around 95% of total UVR reaching the Earth’s surface is UV-A, while only 5% is UV-B [19,41]. It is important to note that phototoxicity increases with decreasing wavelength i.e., UV-B and UV-C are more hazardous than UV-A radiation [42]. DNA damage caused by UVR is represented by cyclobutane pyrimidine dimers (CPD), (6-4) pyrimidine-pyrimidone photoproducts (6-4PP) and oxidative damage [43]. UV-B radiation causes direct damage to DNA while UV-A radiation only causes damage by indirect mechanisms such as increasing oxidative stress [19]. However, solar UVR also plays a beneficial role in humans by increasing vitamin D synthesis in the skin [44]. Interestingly (but paradoxically), it has been suggested that UVR may actually have an anti-cancer effect: in vitro and experimental animal models have both demonstrated that 1,25-dehydroxyvitamin D3, which is formed from vitamin D, inhibits the growth and induces apoptosis of various malignant tumours cells [44].

### 2.2. Transmission of Wavelengths through Ocular Structures

Anatomically, the eye does not have the same susceptibility to UVR as the skin because the eye has physical protective mechanisms that is not present in the skin, including behavioural adaptations, pupillary constriction, wavelength filtering and a protective shadow cast by the upper orbital rim and brow area during high solar angles [41,45]. On the other hand, the eye is designed to focus incoming light rays to form images [46]. Since this requires concentrating the light or increasing the power density of light on the retina and in particular the posterior pole, the internal eye may actually be more susceptible to light damage from the same level of environmental sunlight than skin [46].

The penetration and filtering of UVR differs between the eye and the skin, but in the case of the eye, the filtering of non-visible wavelengths differs substantially by age. In the skin, both UV-A and UV-B radiation can penetrate the dermis; UV-A more deeply than UV-B [19]. Experimental wavelength transmission experiments in normal human and animal eyes ex vivo and in vivo respectively suggest transmission of UV-A (315–400 nm) and UV-B (280–315 nm) radiation to the posterior eye is minimal [47,48,49,50,51]. The adult human cornea completely filters out wavelengths below 280 nm [51], 290 nm [50], 300 nm [47,48] or 320 nm [52]. The amount of light absorbed by the cornea is wavelength dependent. One study found that approximately 70% of 400 nm wavelengths passes through the cornea but transmission decreases to below 20% at wavelengths less than 300 nm, reaching 0% with wavelengths smaller than 290 nm [49]. Similarly, another found that transmission through the centre of the adult cornea decreases from 74% to 27% between wavelengths 400 and 310 nm [53]. The small portion of remaining UV light that passes through the cornea is unlikely to be absorbed by the aqueous humour, which only filters out wavelengths less than 220 nm [47,48] and similarly, the vitreous humour theoretically filters out wavelengths less than 300 nm [47,48] to 280 nm [50]. Ex vivo transmission of UVR (<400 nm) wavelengths through the lens is less than 10% in children and even lower in adults [11,12,47,48] although a small transmission peak have been observed at 320 nm in the lenses of children less than 5 years old [48] and the lenses from people aged 7–19 years [52]. This has been supported in vivo, where young human adults (18–20 year old) were able to detect wavelengths as low as 315 nm to varying extents regardless of eye colour [54].

A general conclusion is therefore that a small proportion of UV light, mainly between 300 nm and 400 nm, reaches the anterior uveal structures throughout life, but a miniscule amount reaches the posterior of the eye, especially in older adulthood. Despite the large proportion of the more damaging UV light being absorbed by the anterior ocular structures, recent papers have presented persuasive evidence of UV light reaching the retina. This neither indicates penetration as far as the choroid nor a dose with carcinogenic potential, but certainly draws into question early reports of complete UVR filtering by the adult eye, and in the context of the light focussing function of the eye, may demonstrate a substantial lifelong UVR dose to the posterior eye: perhaps enough to explain the molecular evidence of UVR damage which has been reported in a proportion of uveal melanoma cases (discussed further in Section 2.4).

### 2.3. The Impact of Age on Transmission of UVR through Ocular Structures

**Figure 1:**
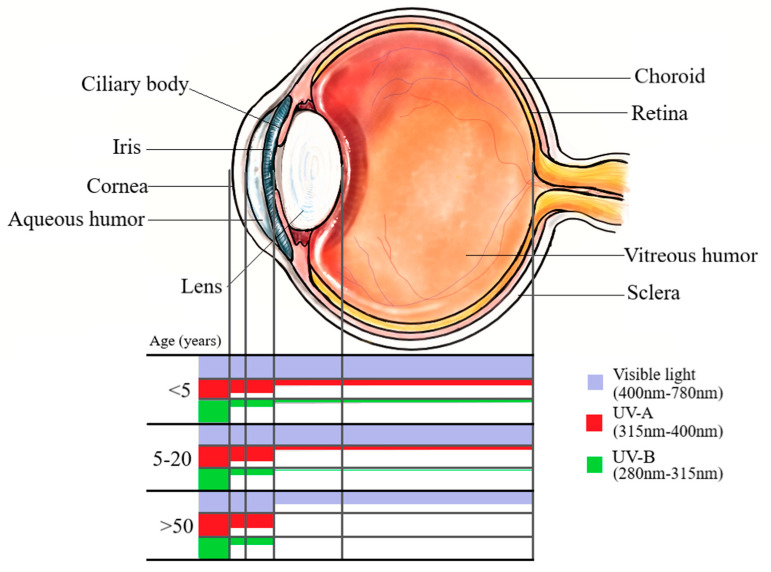
Transmission of UV wavelength spectra through ocular structures at different ages.

Age-related changes to the eye impact UVR transmission substantially. Although exceptions exist [48,55], in general the young eye (less than 20 years) and older eye (greater than 50 years) have differences in wavelength filtration (see Figure 1) and oxidative proteins that certainly impact their exposure to high energy light radiation, and could influence anterior or posterior UM.

In the case of the older eye, a greater lifetime’s cumulative exposure to sunlight is to be expected for any given geographic location, and indeed UM presents most commonly in older age with a peak incidence worldwide from 57 to 70 years old [29,56,57]. Conversely, reports outlined above indicate that the amount of UVR penetrable through to the posterior structures of the eye is negligible after the age of 22 [47]. The older adult lens scatters light more than young lenses [48] and a natural yellowing process of the older human adult lens means that wavelengths less than 400 nm are completely absorbed by approximately the age of 60 [11,48] and even transmission of the visual spectrum from 400 nm to 500 nm is reduced to half at 50 years old and less than 25% at ages greater than 70 [11,55,58]. This theoretically excludes any of the UVR spectrum reaching the posterior of the eye, likely counteracting some elements of what would otherwise be a cumulative lifetime exposure to UVR.

Meanwhile, age-related changes in UVR susceptibility may lead one to expect older adults to have greater anterior (iris or ciliary body) melanomas and less posterior (choroidal) melanomas than individuals under 20 years, but epidemiological data does not reflect this. Ex vivo methods have demonstrated that wavelengths of the UVR spectrum beginning at 300 nm are only able to penetrate the lens to the posterior of the eye in humans aged 30 or less, with increasing transmission of wavelength decreasing age until birth [48,59]. Meanwhile, people greater than 50 years old appear to have increased susceptibility to anterior uveal melanomas. After the age of 50, UVR filters in the cortices and nuclei of normal human lenses become bound to proteins to a significant extent, promoting greater oxidation of proteins after UVR exposure when compared to the cornices of those less than 50 years old [60]. Indeed, UVR exposure increases the risk of other anterior eye diseases such as photokeratitis, photoconjunctivitis, cataract, pterygium and squamous cell carcinoma of the cornea and conjunctiva [46]. Thus, if UVR were complicit in UM aetiology, and the effect does not take many years to manifest, older cohorts would be expected to have greater proportions of anterior melanoma, and less of choroidal melanoma, than younger cohorts. Instead, UM patients 20 years old or younger appear to have a lower burden of choroidal melanomas compared to patients greater than 50 years old: the choroid was the site of origin in only 27.3% [61], 54% [62] or 71% [63] of UM cohorts less than 20 years old compared to making up 90% of cases aged greater than 50 years of age [63]. The higher burden of anterior, instead of posterior, UM in cohorts less than 20 years old compared to patients greater than 50 years old counterindicates a role of UVR in UM.

The theoretical relationship between UVR, age and location of UM origin is only valid if the assumption is that UVR causes fast-acting oncogenic changes to uveal melanocytes. On the other hand, the greater transmission of UVR in younger eyes (less than 20 years old) could merely be a risk factor predisposing to UM development later in life, supporting the peak UM incidence at greater than 50 years old and the greater proportion of posterior UMs in this cohort than people less than 20 years of age. Indeed, other eye diseases seen in older age, such as cataract and macular degeneration, are often advanced stages of a continuous process of deterioration that begins in childhood, suggesting that reduced sunlight exposure in childhood can reduce the incidence of these diseases in adulthood [64]. Childhood sunlight exposure has also been linked to cutaneous melanoma development later in life (e.g., meta-analysis by [65]). Only few studies have assessed odds ratios (ORs) relating to greater childhood exposure to sunlight and development of UM later in life (included in Table 1), but none explore specifically UM cohorts that were greater than 50 years old at time of diagnosis. The presence of freckles in younger age is the main risk factor used as an indication of childhood sunlight exposure [31,33,34]. Two Australian studies agreed that mild childhood freckling increased the risk of developing UM in adulthood compared to no freckling in childhood [33,34], but drew opposing conclusions when heavy childhood freckling was assessed (OR 1.4 [34] and OR 0.88 [33]). A mild (OR > 1 to <1.5) to major (OR ≥ 1.5) risk of adulthood UM development was also seen with childhood freckling (mild to heavy) in a German case-controlled study [31]. Supporting this finding, exposure to artificial UVR (sunlamp use) increased the risk of UM regardless of age, but the risk was greater in those who first used them at less than 20 years old (OR 1.7 to 4.5) than those that first used a sunlamp at greater than 20 years old (OR 1.3 to 1.8) [31]. Greater degrees of squinting in childhood also correlated with mild to major risk of developing UM in adulthood [34], but as greater squinting correlated with a lighter eye colour [34], it is unclear whether the act of squinting was thought to reduce the childhood exposure to sunlight or if the light eye colour increased the child’s susceptibility to developing UM regardless of the amount of squinting. Overall, increased exposure to UVR in childhood, coupled with a greater transmissible UVR spectrum through ocular structures at this age, could point to a greater incidence of UM later in life if UVR is involved in a chronic aetiology, but published data investigating this idea is lacking.

In general, cornea of all ages completely absorbs wavelengths <290 nm. Absorbance by the cornea decreases with increasing wavelength from 290 nm. Age-related changes to the lens allow transmission of >300 nm at less than 5 years old, and ≥315 nm by age 20. Increasing age from 20 to 50 correlates with increasing minimum permissible wavelength (not shown) until minimum permissible wavelength by age >50 years is 400 nm. NB: There is evidence to suggest that blue light (400–500 nm), which is part of the visible spectrum, could cause ocular damage (see Section 6.1)

### 2.4. Effect of Melanin on UVR Susceptibility in the Eye

The quality and quantity of melanin determines the extent of susceptibility to UVR damage in melanocytes and this correlates with UM risk. Having light (blue, gray, green or hazel) coloured eyes as opposed to dark (brown or black) coloured eyes has frequently been found to be a mild to major risk factor for developing UM (see Table 1) in Australia [33,34] and countries in the northern hemisphere [30,32,35]. This could be due to the UVR protective properties of eumelanin in darker eye colours [5]. Eye colour [66,67] is generally based on the quality and quantity of the pigment melanin within melanocytes, and not on differences in the number of melanocytes. There are two main chemically distinct forms of melanin pigments, eumelanin and pheomelanin. It has been observed that in melanocytes from eyes with dark-coloured irises (brown or black), the amount of eumelanin, the ratio of eumelanin:pheomelanin and the total melanin were significantly greater than those from eyes with light-coloured irises (hazel, green or blue) [67]. Eumelanin scavenges free radicals and has superior photoprotective properties than pheomelanin [5]. On the other hand, pheomelanin photodegradation can contribute to DNA damage or apoptosis by generating hydrogen peroxide and superoxide anions [5]. This means that the melanocytes in the eyes of people with light-coloured irises have greater susceptibility to UVR damage, and therefore malignant transformation, than in people with dark-coloured irises. Melanin quality in uveal melanocytes may also change during malignant transformation. A comparison of melanocytes and melanoma cells from light-coloured and dark-coloured irises found that while the melanoma cells shared approximately the same pheomelanin levels as their melanocyte counterparts, UM cells had only 1/8th and 1/31st the eumelanin levels that were in the counterpart melanocytes from eyes with light-coloured irises and dark-coloured irides respectively [68]. Differences in melanin quality in uveal melanocytes could be why populations with very high proportions of Caucasians–an ethnicity generally characterised by light skin and eyes–have much higher incidence of UM than darker ethnicities [25,26]. However, it has also been suggested that light-coloured eyes may have an increased susceptibility to the light spectrum, rather than UVR [69], which could be driving this increase in UM incidence (see Section 6.1). The higher incidence and risk ratios of UM in people with light-coloured eyes compared to those with dark-coloured eyes could be due to the UVR protective properties of eumelanin in the latter, suggesting a possible role of UVR in UM aetiology and progression.

## 3. Molecular Changes in UM in the Context of UVR Influence

### 3.1. Molecular Signatures of UVR Damage in UM

Evidence of UVR damage to DNA depends on how molecular UVR damage signature is defined. UV light induces different types of DNA damage including cyclobutane pyrimidine dimers (CPD) and to a lesser extent, (6-4) pyrimidine-pyrimidone photoproducts (6-4PP) [43]. CPD make up to 85% of all photodamage and are repaired by nucleotide excision repair [43]. Cytosine-containing CPD are highly mutagenic, leading to C:G to T:C transition mutations and CpC:GpG to TpT:ApA tandem transitions, which could be interpreted as a UVR damage signature [43]. This is reflected in CMs, in which UVR plays a significant role. For example, 85% of wild-type *BRAF/NRAS* CM tumours were composed of C > T or G > A transitions, 95% of single nucleotide variants were at dipyrimidine sites and 86% of these were C > T [70]. Similarly, C to T mutations enriched at 3′ positions of pyrimidine dimers were seen in 80–90% of CM mutations in another study of melanomas [71]. The C to T changes seen in ~35% of the 12 UMs investigated were conversely not enriched at the 3′ position of pyrimidine dimers and therefore UVR was not thought to be involved [71]. Meanwhile, Goh et al. compared over 1000 UM mutations and over 12,000 CM samples and found that the proportion of samples with C to T substitutions was higher in UM (17.0%) than CM (13.1%), suggesting a substantial number of UMs are related to UVR damage [6]. A further 103 whole-genome sequencing analyses found that a significant subset of UM characterised by UVR-damage mutations was restricted to those originating from the iris [7], the most anterior structure of the uveal tract and therefore with the greatest exposure to UVR. One of 32 European metastatic UM samples had a distinctive UVR-induced profile based on a dominant signature 7 contribution [8]. Signature 7, mainly found in malignant melanoma, shows a higher prevalence of C to T mutations on the untranscribed strands than transcribed strands, which is consistent with the formation of pyrimidine dimers through UVR damage [72]. This suggests that a very small proportion of UM cases, especially anterior UM, may have been exacerbated by UVR exposure. However, anterior UMs only account for 10% or less of UMs [63], and most UM cases, including those originating from the iris, have a uniquely low mutational burden, with the cancer largely driven by mutations different to CM (see Section 3.2 below).

### 3.2. Common Genetic Mutations in UM are not Associated with UVR Exposure

Larger number of DNA mutations is a common feature of cancers resulting from high exposure to mutagenic agents such as UVR exposure [73]. As such, high mutation loads are often seen in CM due to UVR damage [70,73]. Conversely, UM has a uniquely low mutational burden [7,71]. Aberrant *BRAF*-driven MAPK pathway activity is the dominant oncogenic driver in CMs [74] and is a mutation considered to be associated with UVR damage [75]. This same *BRAF* mutation has sometimes been observed in as high as 40% of UM samples including those arising from the choroid [6,76,77]. On the other hand, 80–96% of UMs possess *GNAQ* or *GNA11* mutations in a mutually exclusive fashion [78,79,80,81], while this occurs in CM approximately 20% of the time [82]. The aberrant *GNAQ* or *GNA11* mutations code subunits for G proteins that activate the MAPK pathway upstream of BRAF [83,84]. Most of the remaining cases harbor activating mutations in a G-protein-coupled receptor (GPCR) or in phospholipase C β4 (PLCB4) [80,85]. These UM-associated gene mutations do not possess the mutagenic nucleotide variations normally associated with the UVR damage signatures [69] described in Section 3.1. Germline mutation of *BAP1,* a major tumour suppressor in numerous human malignancies including UM [86], is rare in CM [87] (see Section 6.2). Other gene mutations that are common to both CM and UM, some of which are suggestive of UVR aetiology [6], have been reported in detail elsewhere [6,82]. UVR exposure has been demonstrated to initiate or progress CM in melanoma-susceptible animal models [88,89,90] but no report of UVR-initiated eye melanoma in an animal model exists. Meanwhile, aggressive eye melanomas in animals have been initiated through the common *GNAQ* mutation without the need for UVR exposure [80,91,92].

Overall, the molecular evidence suggests that the role of UVR exposure in UM compared to CM is very low but not non-existent. Most cases of UM, unlike CM, have a low mutational burden, are driven by *GNAQ* or *GNA11* mutations and often lack UVR nucleotide variations typically seen in UVR damage. However, a minority of UM cases do indeed share the *BRAF* oncogene or the C to T changes near pyrimidine sites often caused by UVR.

## 4. UVR Susceptibility Relating to Environmental Factors

### 4.1. Differences in UVR at Different Latitudes

Exposure to UVR partially depends on the geographical latitude. The average annual UV light reaching the Earth’s surface is greatest in the equatorial regions [93], suggesting that those people living closer to the equator have greater exposure to UVR. This latitudinal UVR influence has been reflected in Australian and New Zealand CM epidemiology [15]. On the other hand, studies of UM have rarely shown such a correlation. In a case-controlled study in New England analysing a cohort of 197 UM patients, it was determined that a southern residence below latitude 40° N for greater than 5 years (i.e., greater UVR) increased the risk of UM compared to long term residence further away from the equator [39], as is theoretically expected if UVR is involved with UM incidence. However, Australian ocular melanoma cases from 1990 to 1998 showed a weak increase, rather than decrease, in incidence across latitude bands from <30° S to >36° S [21]. This was repeated in a study investigating 16 European countries (6673 patients from 1983 to 1994), where incidence rates increased, rather than decreased, with latitudes further away from the equator [25]. This could be attributed to a higher proportion of light-coloured eyes in northern Europe [25,35]. However, an analysis of 2142 ocular melanomas from 1992–2002 in the US, which focused on non-Hispanic whites, revealed a significant increase (4.91- fold) in UM incidence with increasing latitudes (20–22° to 47–48°) [94]. The opposite trend was seen in this population for external (eyelid and conjunctival) ocular melanomas: higher latitude (20–22° to 47–48°) resulted in a 2.48-fold decrease in incidence [94]. This suggests that external, but not internal, ocular melanomas are influenced by UVR. Frequent inverse correlations between proximity to the equator and decreasing UM incidence could indicate a protective role of UVR towards internal ocular melanomas [94]. Increased production of vitamin D and 1,25-dehydroxyvitamin D3 have been proposed as the protective mechanism. The latter has been found to inhibit the growth of and induce apoptosis of various malignant tumours cells in vitro [44]. On the other hand, contradictions in latitude trends in different populations and locations might be skewed by other confounding endogenous and exogenous risk factors (listed in Table 1). Statistical adjustment for geographic area in France did not change the odds ratio estimates for UM risk factors such as light eye colour, fair skin, or propensity to sunburn [32].

### 4.2. Changes in Global UVR Over Time

Changes in global UVR, as caused by changes in the ozone layer, have had worldwide impacts on CM incidence, a cancer often caused by UVR exposure. It is hypothesised if UM is truly influenced by UVR, it would exhibit similar incidence trends to CM over time. Although the stratospheric ozone has been slowly recovering since 2000, decreases in UV-B radiation at the Earth’s surface have not been detected yet because such changes are still masked by varying attenuation of UVR by ozone, clouds, aerosols, and other factors [95]. It is likely that data of UVR-related cancers especially before 2000 would show an increase in incidence, reflective of an increasing UVR. Analyses of cancer data from England and Wales [96], Denmark [22] and Canada [97], found an increase in CM incidence but not in UM. This strongly suggests that UVR does not play a role in UM. However, this could potentially be an indication that ocular sites display different exposure patterns to UVR compared to skin [97], Additionally, in one of these studies there was an increase in the incidence of iris melanomas [22], with the iris the most likely to be influenced by UVR (see Section 2). Overall, changes in incidence over time suggests that changes in global UVR plays a great role in the development of CM, but little to no role in the development of UM.

### 4.3. Differences in UVR Exposure between Urban and Rural Areas

Direct sunlight only partly contributes to ambient UVR [41]. UV light is also partially absorbed by the earth’s atmosphere and reflected to varying extent by land surfaces, oceans, cloud, and snow [16,93]. This scattering accounts for over 50% of ocular UVR exposure under average conditions [41]. In dense urban areas, the increased pollution and built environment can cause absorption and scattering of UV light thereby reducing UVR exposure [16]. Therefore, if UVR influences UM incidence, it may be greater in rural populations than in dense urban populations. This is supported by the fact that living in a rural area may also have a bias in the population for individuals more likely to work in high solar UVR-exposed occupations such as farming or agriculture, and these have also been found to correlate with an increased risk for UM [40,98]. Alternatively, living in townships of higher altitudes, or geographical areas with less cloud cover, could all be associated with increased UVR exposure [99] and possibly therefore increased UM if UVR is a risk factor. UM incidence in the Australian population from 1990–1998 found a 50% higher incidence for rural residents [21], suggesting a correlative relationship between high UVR exposure and UM in this population. However, variations in social and behavioural factors of populations in rural verses urban locations may also account for this finding (see Table 1).

## 5. The Relationship between UVR Exposure and UM in Australia

It is possible that if UVR exposure is involved in UM progression, then Australia, as a large continent extending from the tropics to well into the temperate zones, would share a similar positive correlation between latitude and increasing UVR as has been found in the same continent for CM. In fact, Australia has one of the highest incidences of CM in the world [14,18] due to a culture of outdoor occupations [14,15], high Caucasian population and proximity to the equator. Additionally, Australians have a higher annual UVR burden (20,000–50,000 J/m^2^) than the USA (20,000–30,000 J/m^2^) and Europe (10,000–20,000 J/m^2^) [41] and surface UVR in Australia has continued to rise at all latitudes since the 1970s [16] Despite the implementation of sun-sense campaigns in the early 2000s, this increase in UVR is likely to be the cause of an increase in incidence of CM in Australia, especially amongst older populations [100]. Establishing a scientific comparison of these findings in such a large continent, using data uniquely administered by a single government, is a compelling approach to the controversy.

The relationship between UM incidence and its relationship with UVR exposure has not been analysed in Australia since the early 2000s [21,33,37,38]. The past research on UM in Australia has been summarised in Table 2, and risk ratios associated with UVR exposing factors and UM incidence are listed in Table 1. In general, it has been observed that the Australian population share risk factors relating to UVR that are similar to the rest of the world. This includes a long lifetime UVR exposure, proximity to the equator, older age, occupational UVR exposure, lighter eyes, and susceptibility to burning. It can be extrapolated from the known risk factors that UM in the Australian continent is a disease of the Caucasian population. Little is known about UM incidence in Australian Aboriginals, but it is hypothesised that their predispo sition, like other darker-pigmented ethnicities, may be low [101]. Given that the annual UV index has been increasing, a stronger relationship between UM and UVR exposure would be expected. However, confounding statistical, anatomical, and molecular evidence, as has been discussed throughout this review, suggests that only a minority of cases, if any, are likely to be influenced by UVR exposure in the Australian context.

## 6. Risk Factors other than UVR Exposure that Influences UM Incidence

Demographic trends in UM such as higher incidences in particular locations or ethnicities may not be due to a relationship between UVR susceptibility and UM, but other factors coincidentally, or ubiquitously, present in the populations. Two such theories will be briefly explored: pathological exposure to other wavelength spectrums and germline genetic predisposition in certain populations.

### 6.1. Wavelengths Other Than UVR That Could Cause Ocular Damage

It has been argued that it is not UVR, but rather blue light, that could be causing this higher risk of UM amongst susceptible populations such as welders. The capacity of sunlight to act energetically in its interactions with cellular molecules does not cease simply because the human eye can see it but is in fact inversely related to the wavelength of light via Planck’s constant. The energy of blue light (400–500 nm) photons can easily reach the posterior uveal tract while retaining almost the same energy as UVA, and thus be deleterious to biological structures [11]. Meanwhile, the adult crystalline lens and cornea theoretically prevent the transmission of wavelengths below 400 nm, which encompasses the whole UVR spectrum. In fact, in the elderly, cataractous change even begins to filter blue light from the retina in an extension of the lens’s lifelong expansion of its broadband filtering window. Damage from blue light is thought to arise photochemically from chromophores, such as melanin, retinoids, and lipofuscin, or from the generation of reactive oxygen species in mitochondria [11]. Studies involving the use of a rat model has shown that upon exposure to fluorescent blue light (434 to 475 nm), rats receiving a calcium channel blocker developed ocular melanoma [103]. Similarly, human UM cells in vitro had a significant increase in proliferation when exposed to blue light compared to controls that were not exposed to blue light [104,105], although a comparison of this rate compared to UVR (without blue light) exposure was not explored. This experiment was repeated in an animal xenograft model, demonstrating the ability of blue light to penetrate the posterior of the eye and cause proliferative changes [106]. Laboratory analysis of *GNAQ/11* mutational distributions and light-induced retinal damage markers lead to the suggestion that this mutation in this gene could be related to light, rather than UVR exposure, with a particular subset observed in those with light-coloured eyes [69]. This could mean that the blue light emitted from the screens of electronic devices could be causing more damage than just disrupted melatonin-sleep cycles [107]. Interestingly, a mild increase in UM incidence has been observed in some countries since the 1990s [22,108] although not in others [21]. One may speculate that an increase in UM incidence over time may correlate with the introduction of electronic devices, rather than with rising global UVR. If blue light is involved in UM aetiology, this may manifest in a rise in UM incidence, especially in older populations whose first exposure to modern technology was in childhood. Further investigation of blue light, as opposed to UVR, on UM is beyond the scope of this review, but many of the same suggested approaches to investigating the effect of UVR on UM aetiology can be easily applied to high energy visible light.

### 6.2. Germline/Familial UM

It may be that genetic drift, rather than high UVR exposure, that has provided a higher-than-normal genetic predisposition to developing UM in certain populations. This is hinted at by the evidence that, overwhelmingly, the greatest risks in the northern Australian state of Queensland’s population for UM were found to be a personal history of CM (odds ratio 2.42) and a family history of UM (odds ratio 6.89) [33]. Perhaps this is related to the higher incidence of UM in Australia compared to much of the world. Congenital UM is a very rare presentation [109,110,111], and the only inherited gene mutation that has been so far associated with familial UM is the BRCA1-associated protein 1 (BAP1) gene [112,113]. Examining 66 UM cases further south in New South Wales, Australia from patients under the age of 50 found that two (i.e., 3%) possessed *BAP1* germline mutations [102]. These states represent only a sub-population of the Australian continent, but the incidence of the *BAP1* germline mutation is more or less comparable to results found elsewhere [8,101,114,115,116]. This suggests a common genetic susceptibility in only a small proportion of UM cases. This does not rule out germline genes other than *BAP1* that have not yet been identified as predisposing factors, nor does it rule out the possibility that a mixture of risk factors, such as UVR exposure and genetic disposition, are independently influencing the incidence of UM in a small number of cases.

## 7. Conclusions

It has long been considered unlikely that UVR plays a role in UM. This is due mainly to early knowledge of the filtering properties of the anterior eye, and more recently to a lack of compelling causative evidence despite reports of risk ratios to the contrary. Various studies worldwide have found increased risk of UM in individuals with increased UVR susceptibility such as those with light eye colour, light skin colour and high cumulative lifetime UVR exposure. This is mirrored by a much higher incidence of UM in Caucasian rather than dark-eyed populations. The Australian continent, where high UVR index and exposure already cause a higher incidence of CM than much of the world, has proven to be a useful-but by no means the only-geographical area to model the possibility that UVR, focussed to a point by the optics of the eye, could impact oncogenesis. The fact remains that most UMs originate from the choroid, a posterior (but admittedly more expansive) ocular structure, while the amount of UVR that can penetrate beyond the anterior ocular structures is essentially obsolete in adults, although certainly not in children, and evidence of UVR reaching at least the retina (and presumably its intensity being magnified by focussing) has been demonstrated in those less than 20 years of age. Increases in CM incidence in relation to UVR differences over time or latitude are infrequently reflected in UM statistics. In fact, many studies, especially in the Northern Hemisphere, have actually shown trends to the contrary, where increasing proximity to the equator (and thus greater UVR) is associated with a decrease in UM incidence. This could be skewed by the northern latitudes being the ancestral home of light-eyed ethnicities. However, alternatively, a protective role of UVR against UM has also been suggested. UM has a uniquely low mutational burden and is, unlike CM, associated with *GNAQ/11* mutations at least 80% of the time, which are not typically associated with UVR changes, despite acting on the same MAPK pathway as the common CM oncogene, *BRAF*. Independently of UVR, a small proportion of cases worldwide and in Australia are caused by genetic predisposition due to germline *BAP1* mutation, while risk factors apparently relating to greater light exposure could be related to damage caused by wavelengths of the high energy parts of the visible light, rather than UVR, however in physical terms the distinction in the effect of photons of different wavelength is, by the original definition, a spectrum. However, genomic sequencing data has recently revealed evidence of molecular UVR damage signatures in a proportion of UM cases from both the anterior and posterior uveal structures. Overall, the anatomical, physiological, statistical, and molecular data paints a picture of UVR involvement in a small minority of UM cases. Ironically, the contraindicative evidence of UVR as a driver of UM may not matter in the context of prevention: odds ratio data suggests that sun avoidance measures could reduce the risk of developing UM despite a lack of conclusive evidence that UVR plays a causative role. One point does seem clear though: on the currently available evidence, it seems that, relative to CM, a far greater portion of UM are likely unavoidable.

## Figures and Tables

**Table 1:** UVR-related risk factors associated with UM in the Northern Hemisphere and in Australia.

Risk Factor Relating to UVR Susceptibility	Risk for UM Incidence	Reference
Light (blonde or red) hair colour	-	[30]
	- to +	[31]
	+	[32]
	-	[33]
	-	[34]
Light (Green/gray/hazel/blue) eye colour	+	[30]
	- to ++	[31]
	+ to ++	[32]
	+ to ++	[35]
	- to +	[33]
	++	[34]
Light skin colour	++	[32]
	+ to ++	[31]
	- to +	[33]
	+ to ++	[34]
Burns easily (with little to no tanning)	++	[30]
	+	[32]
	- to +	[33]
	- to +	[34]
≥1 welding burn, sunburn to eye, or snow blindness	++	[30]
	-	[32]
≥5 eye burns	++	[32]
Freckles	-	[30]
	+	[36]
	- to +	[33]
	+	[34]
Takes outdoor sunny vacations	-	[30]
	++	[36]
Leisure time spent mostly outdoors	-	[30]
	+	[36]
	- to +	[37]
High cumulative occupational sun exposure	++	[37]
Large nevi ≥ 1	+	[30]
	+ to ++	[34]
High cumulative exposure to artificial UVR	++	[30]
	++	[32]
	++	[36]
	+ to ++	[31]
	+ to ++	[38]
High cumulative exposure to solar UVR	+	[32]
	+ to ++	[37]
Freckling in childhood	+ to ++	[31]
	- to + ^1^	[33]
	+	[34]
First use of sunlamp (artificial UVR) at <20 years old	++	[31]
Personal history of CM	++	[33]
Family history of UM	++	[33]
Ancestry from more northern latitudes (Northern Hemisphere)	++	[39]
Southern residence below latitude 40° N for >5 years (Northern Hemisphere)	++	[39]
Northern residence above latitude 36° S (Southern Hemisphere)	-	[37]
Northern residence above latitude 30° S for most of life (Southern Hemisphere)	- to ++ ^2^	[33]
Wearing sunglasses more than half to all the time	-	[33]
Wearing prescription glasses or contact lenses	-	[30]
	-	[33]

White boxes represent risk determined from studies of Northern Hemisphere populations and grey boxes represents findings from Australian data. RR, Risk Ratio; OR, Odds Ratio. -, No risk (RR or OR ≤ 1); +, Mild risk (RR or OR > 1 to <1.5); ++ Major risk (RR or OR ≥ 1.5). ^1^ + with 1–100 freckles but-with >100 freckles. ^2^ ++ at 20–30° S but-at ≤20° S.

**Table 2:** Studies on UM in Australian populations.

Title	Year	Data set	Type of Study	Conclusions	Reference
UV light exposure as a risk factor for ocular melanoma in QLD, Australia	1972 to 1996	QLD cases from the QLD Cancer Registry and from pathology laboratory ocular specimens in QLD 216 choroidal 35 ciliary body 27 iris 35 conjunctival	Determining risk factors using case-control study (125 patients, 375 controls), questionnaire without disclosing study hypotheses OR and 95% CI	No correlation between estimated lifetime cumulative solar radiation exposure and ocular melanoma in QLD from 1972 to 1996 but a protective effect of dark skin, brown eyes, and resistance to sunburn. A family history of ocular melanoma was a strong risk factor.	[33]
Eye colour and cutaneous nevi predict risk of ocular melanoma in Australia	1996 to 1998	NSW, VIC, QLD and Other from “all ophthalmologists and population-based cancer registries in Australia” 222 choroid 22 ciliary body 1 ciliochoroidal 25 iris 19 conjunctival	Determining risk factors using case-control study (290 patients, 914 controls), questionnaire without disclosing study hypotheses OR and 95% CI using STATA and Mantel-Haenszel	Light eyes, cutaneous nevi and inability to tan were found to be risk factors for UM.	[34]
Sun exposure predicts risk of ocular melanoma in Australia	As above	Same dataset as above	Same model as above	Long hours outdoors increase risk of UM.	[37]
Artificial UV light radiation and ocular melanoma in Australia	As above	Same dataset as above	Same model as above, but with 290 patients and 893 controls	Risk of ciliary body and choroidal (but not iris or conjunctival) melanomas increased with exposure to sunlamps or welding independent of personal sun exposure.	[38]
Incidence of ocular melanoma in Australia from 1990 to 1998	1990 to 1998	From Australian population-based cancer registries and all practising ophthalmologists 1990 to 1995 = 768 1996 to 1998 = 539 (choroid or ciliary body 459, iris 42, conjunctiva 37)	Determine the incidence and incidence trends of ocular melanoma based on distribution, latitude and in subpopulations. Incidence trends only used cancer registry-reported cases and excluded QLD	Incidence increased weakly across latitude bands from <30°S to >36°S. Rural residence increased incidence by 50%.	[21]
Survival from UM in WA 1981–2005	1981 to 2005	WA Cancer Registry 229 Choroid 33 Ciliary 46 Iris or Unknown	Determining relative survival estimation and proportional hazards regression models using STATA Eerer II methodology(308 patients)	When life tables were used to account for the baseline death rates in the general population, the relative survival rates at 3, 5 and 10 years were 88% (95% CI: 83–92%), 81% (95% CI: 76–87%) and 71% (95% CI: 63–78%), respectively	[57]
Prevalence of germline *BAP1* mutation in a population-based sample of UM cases	(Not specified) 2012 or earlier based on publication date	NSW Patients diagnosed at ≤50 years old (*n* = 66)	Sanger sequencing to determine *BAP1* mutations	2/66 possessed *BAP1* mutations	[102]

QLD, Queensland; VIC, Victoria; NSW, New South Wales; WA, Western Australia.
